# Environmental allergen reduction in asthma management: an overview

**DOI:** 10.3389/falgy.2023.1229238

**Published:** 2023-10-06

**Authors:** Duy Le Pham, Kieu-Minh Le, Diem D. K. Truong, Huyen T. T. Le, Tu H. K. Trinh

**Affiliations:** ^1^ Faculty of Medicine, University of Medicine and Pharmacy at Ho Chi Minh City, Ho Chi Minh City, Vietnam; ^2^ University Medical Center Ho Chi Minh City, Ho Chi Minh City, Vietnam; ^3^ Center for Molecular Biomedicine, University of Medicine and Pharmacy at Ho Chi Minh City, Ho Chi Minh City, Vietnam; ^4^ University of Medicine and Pharmacy at Ho Chi Minh City, Ho Chi Minh City, Vietnam

**Keywords:** asthma, allergen, aeroallergen, severe asthma, asthma management

## Abstract

Asthma is a prevalent non-communicable disease that affects both children and adults. Many patients with severe, uncontrolled asthma could not achieve total control despite using anti-asthmatic drugs. There is increasing evidence that allergy to environmental allergens, including both indoor and outdoor allergens, is associated with asthma symptoms and severe asthma. Frequently reported sensitized allergens were dust mites, cockroaches, grass pollens, molds, pets, and rodents in allergic asthma patients, although the patterns of widespread allergens differed from each country. Allergen avoidance is the cornerstone of asthma management, especially in sensitized subjects. This review summarizes environmental allergen avoidance and clarifies their effects on asthma control. Despite contrasting results about the impact of allergen exposure reduction on asthma control, several studies supported the beneficial effects of reducing asthma-related symptoms or risk of exacerbations as a nondrug therapy. Identifying environmental allergens is helpful for asthma patients, and further studies on clinically effective avoidance methods are required.

## 1. Introduction

Asthma is one of the most prevalent chronic, non-communicable diseases; global prevalence estimates were 9.8%–17.9% (1). The hallmarks of asthma are chronic airway inflammation, leading to recurrent respiratory symptoms, and variable expiratory airflow limitation (2). The pathogenesis of asthma is complicated by complex gene-environment interactions, leading to heterogeneity in clinical presentation. Initially, asthma was classified as non-atopic or “intrinsic” asthma and atopic or “extrinsic” asthma. Recently, asthma endotypes are divided into type 2 (T2) high or T2-low (3–5). The T2-high atopic asthma usually manifests as early onset, steroid sensitive and allergic sensitization with positive allergy skin tests and increased serum-specific IgE. Allergic asthma is predominant in early childhood, which gradually declines with advancing age. In contrast, most patients with asthma onset after 40 years of age are non-allergic (6).

Pharmacological treatment of asthma is based on anti-inflammatory drugs and bronchodilators to achieve asthma control, i.e., to reduce symptoms and prevent exacerbations (2). Despite this, some patients could not achieve complete asthma control, defined as poorly controlled or severe asthma. Individuals with severe asthma were frequently older and presented more symptoms, accompanied by limited activities and lower lung function (7). In 2019, more than 260 million people had poorly controlled asthma (8). Additionally, asthma is responsible for over 1,000 deaths a day (9–11). The rising trend of asthma leads to an increased burden on patients’ quality of life and economic burden. Asthma significantly affects patients’ quality of life physically and emotionally, in both adults and children (12, 13). The quality of life was negatively worse in patients with severe asthma (14, 15). In the United States, asthma costs increased from $53 billion to $56 billion within five years from 2002 to 2007, reaching $81.9 billion in 2013 (16, 17).

There is a need to improve asthma control and management. Multiple factors were shown to be associated with uncontrolled asthma, including allergen sensitization, socioeconomic status, and climate changes (18–21). Growing evidence shows that allergy is essential in asthma pathogenesis, particularly severe asthma, in both children and adults (7, 19, 22–25). Therefore, the identification of allergens triggering asthma symptoms may be potential to improve disease control. Generally, environmental allergens are classified as indoor allergens (house dust mites, pet dander, mold, cockroach, rodents) and outdoor allergens (mold, pollens), which would be later discussed. Low socioeconomic status (SES) has been shown to be related to worse asthma outcome as well as being a risk factor for indoor allergens sensitization (18, 26). Additionally, climate change negatively impacts pollen allergy and mold allergy (20, 27). Thus, in the context of this review, we aimed to summarize the current understanding of allergens, particularly environmental allergens, and their association with asthma. Furthermore, up-to-date evidence about means of allergen avoidance in asthma control will be subsequently discussed.

## 2. Overview of environmental allergens

Allergic asthma is one of the most common manifestations of reactions to indoor and outdoor allergens. The main sources of outdoor pollutants worldwide are fuel combustion from vehicular transportation, construction, agricultural operations, power plants, and industries (28). However, indoor environments also pose significant health risks, given that the majority of people spend more than 90% of their time indoors (29). Factors such as building systems, construction techniques, contaminant sources, and occupants’ behavior can affect the indoor environment. The most commonly studied risk factors for indoor pollution are environmental tobacco smoke, biomass fuel, cleaning products, and biological allergens (30). House dust mites (HDM), furred pets (primarily cat and dog dander), cockroaches, molds, plants, and rodents are the main sources of indoor allergens (31, 32). Main outdoor allergenic sources include plants (pollen, fern spores, soy dust) mold (spores, hyphae), and yeasts (33). The significant rise in allergy incidence in recent decades cannot be solely explained by genetic factors but by increasing air pollution, changing lifestyles, and interactions between biological allergens and air pollution (34, 35).

There is conflicting evidence regarding association between early exposure to mite allergen and asthma development (36). Symptoms of dust mite allergy in asthmatic children (37) and among adult asthmatics that are mite sensitive, poor pulmonary function, and aberrant bronchial reactivity (38) all correlate with the mite allergen level in their home. Seasonal changes in dust mite allergen exposure result in seasonal changes in bronchial hyperreactivity (39). According to a study on dust from primary health care centers (PHCCs) in Lisbon, Portugal in 2018, levels of dust mite Der p 1 and Fel d 1 ranged from 13.0 µg/g to 971.0 µg/g and from 7.0 µg/g to 4618.8 µg/g, respectively (40). Bases on a few studies investigated the presence of household mites in several cities in Brazil in 1998, elevated levels of Der f 1 were found in the beds of asthmatic patients (15.8 μg/g dust) and non-asthmatics (8.2 μg/g dust) (41). On the other hands, Der p 1 levels were lower in the beds of these same individuals (asthmatics—2.8 μg/g dust; non-asthmatics—4.9 μg/g dust) (41). Samples were collected from surfaces such as sofas and beds (including mattresses, bedspreads, and pillows) in 60 households during two separate periods (March and July) (42). Higher levels were found in March in these samples; Der f 1 levels were 31.7 μg/g dust in beds and 8.3 μg/g dust on sofas (42). In a US birth-cohort study of 440 children, early exposure to dust mite allergens at levels of ≥10 µg/g was linked to an increased risk of asthma at age 7 (43). Another study in Taiwan children in 2011 showed that early-life exposure to carpet at home was associated with early-onset asthma and ever-having asthma, particularly when the carpet was in the child’s bedroom (44). Additionally, an ISAAC study of 6,928 Chinese schoolchildren aged 13–14 years in 2009 found that increasing sensitization to dust mites was associated with an increased prevalence of wheezing, with high degrees of sensitization serving as a risk factor for asthma diagnosis (45).

Cockroach, dogs, cats and rodents are important indoor allergens. Dust mite allergen (Der f 1 and Der p 1), cockroach allergen (Bla g 1), cat allergen (Fel d 1), and dog allergen (Can f 1) with concentrations of 10 μg/g, 8 Units/g, 8 μg/g, and 10 μg/g, respectively, are asthma symptom thresholds for sensitive children. Additionally, the kitchen has 1.6 μg/g quantities of mouse allergen (Mus m 1) (45–50). According to a study conducted in Taiwan, exposure to dogs or any pets during early life was significantly associated with the onset of asthma before age 5 (44). Regarding rodents, a study from Netherlands reported the geometric mean of the mouse allergens and rat allergens in settled dust in positive houses was 2.5 ng/m^2^ (GSD 3.6) and 39.3 ng/m^2^, respectively (48). Additionally, mouse allergens were detectable in inner city homes of children with asthma (49).

Airborne fungi are both indoor and outdoor allergens, with major indoor fungi are *Penicillium*, *Cladosporium*, *Aspergillus* and outdoor species are *Cladosporium*, *Penicillium*, *Aspergillus*, and *Alternaria* (50). This study also found that exposure to mold odor during early life was associated with the development of asthma after 5 years of age, while visible mold exposure during this period was linked to ever having asthma (44). Building dampness and molds have also been linked to respiratory and asthma-related health outcomes, with specific molds such as *Aspergillus* and *Penicillium* shown to increase the risk of current asthma in children (51).

In terms of pollen—an important source of outdoor allergens, pollen counts can vary significantly throughout the day and be influenced by weather conditions in short time intervals (52, 53). Consequently, people who suffer from allergies often monitor published pollen counts and modify their behavior based on these fluctuations. A particular study revealed that a greater quantity of total pollen was collected at a height of 1.5 meters above the ground (25,204 grains) compared to 35 m (16,218 grains) or 70 m (14,408 grains) (52–54). A study involving 98 children diagnosed with allergic rhinitis and asthma found that nasal scores exhibited a linear increase in relation to pollen counts, ranging from 0 to 30 grains/m^3^ (55). Subsequently, symptoms escalated at a quicker pace until the counts reached 80 grains/m^3^ (55). Temperature and precipitation were demonstrated to affect the airborne levels of pollen and mold spores (56).

Children and teenagers in population-based research in southern Vietnam showed a high incidence of cockroach and house dust mite sensitivity, but much lower rates of sensitivity to mold and pollen (57, 58). In the south of Vietnam, dust mites and cockroaches were the most common allergen sensitizers among patients with chronic respiratory diseases. That relocation from rural to urban areas increased the incidence of dust mite sensitization (59). Our current study demonstrated that house dust mite [*Dermatophagoides farinae* (Df)*, Dermatophagoides pteronyssinus* (Dp), *Blomica tropicalis* (Bt)] remains major allergens in adult asthma as well as pediatric asthma in Vietnam (unpublished data). Thus, inhalant allergens are major allergens to patients with respiratory diseases, including asthma, and means of allergen prevention can benefit asthma control.

## 3. Association of environmental allergens with asthma control

Many factors, such as adherence, intrinsic factors, and environmental exposures, contribute to poor asthma control (60). Various studies demonstrated the associations between different types of allergens, including outdoor and indoor allergens, with the development and severity of allergic diseases, including asthma (61, 62), and lack of control of severe asthma was positively correlated to the co-existence of moderate-to-severe rhinitis (63, 64). A cross-sectional study in 12,743 patients showed the ratio of the uncontrolled group was 34.71% 8,517 patients had a history of allergies (64). Allergen exposures increased the risk of asthma exacerbation and the development of fixed airflow limitation (64).

Several studies demonstrated that the inner-city home environment characterized by high airborne pollutants and higher levels of mice and cockroach were associated with more significant asthma morbidity (65–67). HDM Der p 1 allergens were proven as an independent trigger of asthma, and pet allergen levels were associated with greater asthma severity (68). Pet allergen exposure was also related to difficult-controlled asthma (69). In Table 1, we summarized the studies demonstrating the association between environmental allergens with respiratory diseases, including asthma and other allergic diseases related to asthma. Atopic sensitization is a risk factor for asthma, and the interaction between rhinovirus and high titer IgE, especially with dust mites, enhanced the risk of asthma exacerbation (70, 71). Fungal sensitization, particularly *Aspergillus,* was associated with asthma symptoms, increased risk of exacerbations, and severe asthma (19, 22, 23). There is increasing evidence about the relationship between indoor dampness and visible mold, especially mold odor, with the risk of the onset of asthma (72). A suitable environment, including temperature, substrate, and high indoor humidity level or water damage, is necessary for indoor mold growth, leading to increased spores of some species that can cause asthma and exacerbate symptoms (73, 74). Outdoor fungal exposure, especially *Alternaria* and *Cladosporium*, was associated with persistent, severe asthma and the severity of asthma exacerbations (75–78). Children with asthma usually have comorbid allergic diseases, with severe rhinitis may be a significant driver of severe asthma. Studies also demonstrated the association between severe asthma exacerbation with allergies to foods, pollen, and pets (24). Especially cats and dogs are assumed to enhance endotoxin-associated asthma and wheezing (79, 80).

**Table 1:** Characteristics of environmental allergens in asthma patients.

Country	Study design	Main findings	Ref
Vietnam	423 patients	Mites and cockroach were the most prevalent sensitizing allergens: Df (59.8%), Dp (50.4%), Bt (49.6%), storage mites mix (10.4%), and cockroach (10.2%)	(81)
	610 CRD patients, 56% had chronic obstructive pulmonary disease and 31% were asthma patients	50% of asthma patients were sensitised to mites and 70% were sensitised to at least one airborne allergen. The most frequent sensitisers were dust mites (Df 22%, Bt 19%, Dp 18%) and cockroach droppings (13%).	(59)
	684 patients based on modified GA_2_LEN study questionnaire	The most common sensitizer among adults in northern Vietnam was the storage mite Bt (men 27.7%; women 18.7%), followed by Dp (men 16.5%; women 10.6%); and Df (men 15.3%; women 6.3%), and cockroach (men 16.5%; women 10.2%).	(82)
Africa	Systematic review: 20 studies from 1967 to 2018	The prevalence of fungal sensitisation was relatively high (3%–52%) in the asthmatic population with an average of 28% and a pooled estimate of 23.3%, mostly due to *Aspergillus* species	(22)
USA	Settled dust samples were vacuumed from 87 classrooms	Mouse allergen was detectable in 81% of the samples collected. Cockroach allergen (Bla g 2) ranged from below limit of detection (<0.003 microg/g) to 1.1 microg/g. Cockroach allergen was detected (>0.003 microg/g) in 71% of the dust samples. Bla g 2 was detected in 22% of airborne samples from the schools. Mouse allergen was only detected in 5%.	(83)
	127 physician-diagnosed asthma children	26% were sensitized to mice. mouse-sensitized children exposed to higher levels of Mus m 1 (>0.5 microg/g) had 50% more days of symptoms and 80% more days of beta-agonist use than others.	(84)
UK	121 severe asthma patients	87/121 subjects sensitized to one or more fungi. In single fungal sensitive group, 45% sensitized to *Aspergillus*, followed by *C.Albicans.*	(85)

Bt, Blomia tropicalis; C.Albicans, Candida Albicans; CRD, Chronic Respiratory Diseases; Dp, Dermatophagoides pteronyssinus; Df, Dermatophagoides farinae; GA_2_LEN, The Global Allergy and Asthma European Network.

Notably, the allergens patterns could vary between studies depending on climate, humidity, and geography. Low SES and climate change could play detrimental effects on airborne allergen levels. Asthmatic patients with a lower SES were more likely to engage in poor health behaviors, including higher rates of smoking, obesity, a higher number of pack-years, and excessive dietary fat intake (18, 86). Furthermore, low SES patients may have higher exposures to some allergens, as studies have shown the increased cockroach and mouse allergens levels in the houses where subjects with low SES lived in (87–89). As discussed above, climate change variables average weather conditions such as global warming, air pollution from traffic and industry, and more frequent extreme climate events effected in dampness proliferation of molds, pollen and fungi concentrations, which were suggested to be related to exacerbation rate, morbidity, and mortality of asthma and respiratory diseases (90–92).

Compared to Western countries, the profile of allergen sensitization in Eastern countries was slightly different (Table 1). Given the close relationship between allergens and asthma, measures to reduce the impact of environmental allergens are essential to lessen the severity of asthma in susceptible patients (93).

## 4. Measures of environmental allergen controls

### 4.1. House dust mite

House dust mites (HDMs) are associated with an increased prevalence of perennial allergic rhinitis at lower concentrations and asthma at higher concentrations (94). The main HDM species include *Dermatophagoides farinae, Dermatophagoides pteronyssinus, Euroglyphus maynei,* and *Blomia tropicalis.* The most significant concentration of HDMs is detected in bedding, especially with increasing temperature and humidity during sleep, in fabric-covered furniture, carpets, and soft toys, as these areas provide an ideal food source in the form of exfoliated human skin (95). There have been numerous proposed measures to avoid dust mites, such as using mattress and pillow encasings, employing high-efficiency particulate air filtration (HEPA) vacuum cleaners, utilizing air purification systems, employing acaricides, controlling humidity, and physically removing mite reservoirs:

- *Usage of suitable materials for covering pillows and mattresses:* Fine woven fabrics with a pore size of less than 6–10 mm are recommended for pillow and mattress covers. Front-loading washing machines, which do not completely submerge goods in water, are less successful than toploading washing machines at removing mites from blankets when using cold water (96). To eradicate dust mites from blankets, use hot water in a front-loading, high-efficiency washing machine (96). All other materials in the bed should be regularly washed and dried to kill mites. The use of detergent, bleach, and repeated washing can also be important (97, 98).
- *Humidity control:* Lowering humidity levels can effectively inhibit dust mite growth; however, it is essential to maintain a consistent relative humidity well below 50% for this approach to be successful (61). The effectiveness of different methods to achieve such humidity levels varies depending on house design and climate conditions. In regions with temperate climates and high relative humidity, portable dehumidifiers have not demonstrated any significant impact on the presence of dust mites or the levels of mite allergens, although air conditioning and central dehumidification capable of lowering humidity below 50% can be of benefit (99).
- *Use of vacuum cleaners with (high-efficiency particulate air cleanser) HEPA filters:* To reduce exposure to dust mite allergen-containing particles, it is advised to regularly vacuum using cleaners equipped with HEPA filtration or opt for a central vacuum system with sufficient filtration that vents to the outside (100, 101). It is recommended to use vacuum cleaners equipped with a HEPA filtration system to trap dust mite fecal pellets effectively within the vacuum bag to remove HDMs from carpets and furniture (100, 101). While HEPA filters may not be highly effective for reducing airborne mite exposure due to the fact that mite allergens only become airborne briefly following disturbance of a soft substrate, they can still be beneficial in specific situations, although less so for mite allergens than for the smaller particles of animal dander allergens (102).
- *Cleaning carpets and upholstered furniture:* The majority of mite avoidance strategies were designed with the assumption that individual patients primarily encounter house dust mites (HDM) during their time spent in bed, on the floor, or on upholstered furniture (99). However, it is now suggested that for younger children who spend the majority of their time at home, with more hours spent in bed or on the floor than adults, the main mite reservoirs and sources of exposure might be mattresses and carpets (103). On the other hand, other sources of HDM might have a significant impact on adults’ exposure to these allergens (103). If possible, it is best to remove carpeting, especially in the bedroom, leaving a smooth wood or other wipeable floor. Acaricides applied to carpets have not shown clinical benefit. As mentioned, vacuuming with a vacuum cleaner with a HEPA will remove some of the mite allergen, but it cannot remove the mites themselves (99).

### 4.2. Cockroach

The two most frequently encountered cockroach species in domestic homes and public buildings are *Blatella germanica* (German cockroach) and *Periplaneta americana* (American cockroach). When disrupted, cockroach protein, similar to dust mite allergen, becomes airborne and settles down rapidly (104). Although most studies on cockroaches and asthma have focused on urban areas, cockroaches can also infest buildings in suburban and rural locations, regardless of the socioeconomic status of their occupants (105). The main allergens associated with cockroaches, namely *Per a1*, *Bla g1*, and *Bla g2*, have been found commonly in floor dust, as well as in kitchen cabinets, bathrooms, and basements (106). To reduce cockroach allergens, measures such as blocking entry points, eliminating sources of food, water, and shelter, and using traps and insecticides can be taken (43). HEPA vacuuming and covering mattresses can also help eliminate reservoirs of allergens (43). Gel bait insecticides, particularly those containing fipronil or indoxacarb, and hiring a professional pest control service have been proven effective interventions, while sprays should be avoided (107).

### 4.3. Grass

Grass pollens have been classified into 8 classes based on their immunologic properties and contain 20–40 distinct antigens (108). Recent studies suggest that grass pollens are the primary cause of allergic reactions, as they can trigger the rupture of pollen grains, releasing hundreds of toxic starch granules that are small enough (<3 µm) to penetrate and disturb small airways (109). Group I allergens are particularly relevant, as they provoke a reaction in 90%–95% of grass pollen-allergic patients during skin testing (110). Exposure to outdoor tree and grass pollens and fungal spores has been linked to increased allergic illnesses, including asthma (111). Although controlling outdoor pollen levels is challenging, reducing exposure can be achieved by spending less time outdoors, mainly when counts are high and in the morning (112). Besides that, indoor incursion of outdoor allergens can be reduced by closing windows, using air conditioning and high-efficiency particulate arrestor filters, and frequently washing surfaces (112). Additionally, those who are allergic to grass should avoid mowing pollinating grass. Reductions in allergy symptoms have been noticed by employing respirators, goggles, or face masks outdoors (113, 114).

### 4.4. Molds

Molds can grow and spread in both indoor and outdoor environments. There are several common types of molds, including *Cladosporium* species, basidiospores, spores of the *Penicillium/Aspergillus* type, and *Alternaria spp* (25). Most mold allergens are encountered by inhaling mold spores of varying sizes and shapes, ranging from 2 to 250 mm, with many being respirable (115). Floods, leaks, condensation, and household molds are primary indoor sources of mold growth (116). Additionally, molds can grow in aeration/conditioning ducts and water pipes (116). Reducing indoor mold exposure is essential to asthma management in sensitized patients. These interventions may include removing mold from hard surfaces, preventing rainwater intrusion, installing ventilation systems in areas such as attics, kitchens, laundry rooms, and basements, repairing plumbing leaks and water seepage by filling gaps and cracks around the foundation, cleaning exterior guttering, and so on (80). Control of indoor relative humidity under 50% by increasing ventilation and covering cold surfaces, washing with detergent, contaminated carpets and removing wallpaper can be taken to discourage mold growth and mold remediation (117). In addition, the US National Institute of Occupational Safety and Health (NIOSH) recommends using an N-95 mask or higher when removing visible mold, while the US Environmental Protection Agency (EPA) advises seeking assistance from an experienced contractor for mold removal (118). Furthermore, building materials that are extensively contaminated with mold should be replaced. While frequent vacuuming may help lower mold spores in dust, replacing rugs with alternative flooring options appears to be more effective (119).

### 4.5. Pets and rodents (cats, dogs, mice)

The major allergen sources from animals include dander (desquamated epithelium), saliva, urine, hair, and feathers, which produce allergens unique to each species. The main source of cat allergen, especially *Fel d1*, is cat saliva, also in the sebaceous glands and urine of male cats (117). It can be transported through the air by particles larger than 2.5 µm and can remain airborne for long periods (117). Besides, dog allergen, particularly *Can f1* and *Can f2*, is present in dander, saliva, urine, and serum (117). In addition, sensitivity to rodent dander may develop in laboratory workers due to occupational exposure. Still, but the allergenic protein in rodent urine can also contribute to allergic reactions in homes infested with rodents Once asthma is established and sensitization is confirmed, the current general approach is to avoid exposure by removing pets from the home. However, it may take several months (20–24 weeks) to to significantly reduce allergen levels once the pets are removed (120). When the air cleaner was used with the cat in the room, the quantity of the Inhaled *Fel d 1* was reduced by up to one-third (121). Several studies demonstrated that air filtration and mechanical washing with detergents also effectively remove cat and dog allergens and indoor air particles (102, 122). Moreover, positive results were obtained from investigating the effectiveness of using a HEPA air cleaner to reduce airborne cat allergens (121). It can be challenging to avoid exposure to cat allergens as they can be present in public places, around pet owners, and even at school. Thus, it is vital to have comprehensive knowledge of potential exposures to other furry animals. Although avoiding the animal is the best option if there is a proven relationship between exposure and symptoms, it is often not feasible and can have a significant emotional impact (117).

## 5. Effectiveness of environmental allergen avoidance on asthma control

### 5.1. House dust mite

Various methods for HDM avoidance have been introduced; however, the effectiveness of those measures is still controversial.

While allergen immunotherapy to HDM was recommended in managing uncontrolled asthma, most meta-analyses found that HDM control in the living environment did not affect asthmatic management or prevention (123–127). Consequently, HDM avoidance was not broadly recommended in the guidelines for asthma management, including Global Initiative For Asthma (GINA) (128). Nevertheless, several considerations should be taken when analyzing the systematic review and meta-analysis data. The combination of different study populations (e.g., adults and children, HDM avoidance measures (e.g., single and multiple methods), period of intervention (e.g., long and short periods), outcomes (e.g., asthmatic symptoms, hospitalization, lung function, quality of life, etc.) in meta-analyses could affect the final result (129, 130). Notably, clinical trials available in the literature were predominantly conducted in mild-to-moderate asthma and/or well-controlled asthma patients, which could blunt the effectiveness of the allergen avoidance interventions.

In adult asthmatic patients, the use of impermeable bed covers and/or pillow covers as the only intervention was found to be not effective in preventing allergic symptoms, suggesting the need for a multi-faceted approach. In a clinical trial with 1,122 adult asthmatic patients, although the concentration of HDM in mattress dust was lower in the intervention group compared to the control group, morning peak expiratory flow rate, mean reduction in steroid dose, symptom scores and quality of life were not significantly different between the two study groups (131). Similar findings were demonstrated in other clinical trials (132, 133). Another study involving adult patients with allergic rhinitis and HDM sensitization also found that using bed encasing had no effect on improving rhinitis-specific visual analog scale as well as mean daily symptom score (134). Notably, two aspects need to be considered simultaneously when conducting studies to investigate the effects of the allergen avoidance methods: the reduction of allergen concentration and the improvement of respiratory allergy symptoms (135). Avoidance measures that fail to reduce allergen levels obviously cannot be expected to reduce allergy symptoms.

In contrast, studies in children with asthma have showed different findings. A randomized trial in 241 children aged 3–17 with asthma demonstrated that using impermeable bedcover reduced HDM concentration in mattress dust and the prevalence of hospital administration due to severe asthma exacerbation (71). In another study, mattress, and pillow encasings also reduced HDM allergen concentrations and the dose of inhaled steroids used by asthmatic patients (136). A multifaceted home-based intervention, including the use of allergen-proof bedcovers and fragrance-free cleaning products as well as avoidance of indoor smoking, showed a significant reduction in daily asthma symptoms, nighttime awakening, and steroid use in children with asthma (137). Other studies in children also found that control of HDM allergens by using single or multiple methods could provide a beneficial effect on asthma management (138–140).

Interestingly, a recent study found that staying at home during COVID-19 pandemic improved asthma control and reduced inhaled corticosteroid use in children with asthma and HDM sensitization (141). This finding suggests that air pollution could be an important confounding factor to consider when investigating the effectiveness of in-door allergen avoidance.

### 5.2. Cockroach

Interventions in most clinical trials were successful in reducing indoor cockroach allergen; however, the effectiveness in asthma management was controversial. Wood et al. (142) demonstrated that utilization of abamectin and professional house cleaning significantly reduced Bla g1 amount in house dusts; however, the concentration was still above 8 U/g (the level associated with asthma symptoms). Results from the Inner-City Asthma Study (ICAS) demonstrated that interventions to concomitantly eliminate multiple indoor allergens (including HDM, cockroach, pet, mold) and tobacco smoke significantly reduced Der f1, Fel d1, and Bla g1 levels, which was associated with a reduction in asthmatic symptoms and morbidity for 2 years of the study (143). Another study showed that insecticide bait application significantly reduced number of cockroaches, which was associated with a lower asthma morbidity in children living in these houses (144).

In contrast, a clinical trial with multifaceted interventions significantly reduced the levels of indoor allergens; however, the cockroach allergen level (Bla g2) reduction was similar in control and intervention groups (145). In National Cooperative Inner-City Asthma Study (NCICAS), cleaning house and insecticide utilization significantly reduced cockroach allergen Bla g1 level for 2 months, but the allergen levels were increased to the baseline level at the end of the study. The compliance of the study subjects with the cleaning instructions was likely poor (146).

### 5.3. Grass

Besides grass pollen immunotherapy, grass pollen avoidance is highlighted for asthma management to prevent exacerbation (112–114). Due to the limited time window of pollen peak occurrences, susceptible individuals should be advised to stay home and avoid outdoor exposure (1, 2). However, there is inconclusive evidence on clinical outcomes. Masks effectively prevent pollen exposure and reduce nasal and conjunctival symptoms (147). The upper respiratory tract is closely related to asthma symptoms (148). In a meta-analysis, the summary effect estimates for a 10 grains/m^3^ increase in pollen exposure demonstrated a 2% increase in the risk of any allergic or asthmatic symptoms, 1%, 7%, and 11% increase in the risk of lower respiratory symptoms, upper respiratory symptoms, and ocular symptoms, respectively (3). Thus, even short-term pollen exposure increased the risks of asthma symptoms (3). There is a need for studies to personalize the exposure assessment to pollen with asthma control. The AAAAI Aerobiology Committee developed guidelines for hypoallergenic landscape plant selection in pollen-related allergic patients (4). Given that the grass pollen differs from each area depending on the local ecosystem, further studies in various geographical regions are necessary.

Grass avoidance is stressed in specific cases where the seasonal change is closely related to allergic and asthma symptoms. Epidemic thunderstorm asthma is characterized by acute asthma triggered after a thunderstorm (5–7). The incidences of respiratory admissions have been linked to the concentration of airborne allergens in the atmosphere, in association with environmental factors such as rainfall, temperature, and aerosols (5, 8). To prevent epidemic thunderstorm asthma, thunderstorm forecasts, and pollen counts are major factors in issuing public health warnings (7, 8). A case report showed cross-reactivity between Bermuda grass pollen with multiple grains (9). Aside from pharmacological therapy for grass-allergic patients, measures to prevent grass pollen exposure remain mandatory.

### 5.4. Molds

Outdoor allergens sensitive patients should limit outdoor activities in high levels seasons (149). In a systematic review including 12 studies with 8,028 participants, the intervention group with repairing houses or mold-damaged offices showed a reduction in asthmatic and respiratory symptoms compared to the control group in adults and children. However, the evidence was moderate to very low-quality (150). In another study, asthmatic children living in a home with indoor mold were distributed into the remediation group and control group. The remediation group received household repairs (e.g., water infiltration reduction, water-damaged building materials removals, and alterations of heating/ventilation/air-conditioning), while the control group was educated about home cleaning. Although there were no changes in total and outdoor mold indices at the end of the study, children in the remediation group significantly reduced symptoms days and decreased exacerbations compared with control asthmatics (151). Similarly, a global allergen avoidance method conducted by an indoor environment counselor enhanced patients’ lung function, and reduced the number of asthma-related hospitalizations, and the use of anti-asthmatic medication (152). Given the close association between fungal exposure and severe asthma, methods to avoid molds should be noted for vulnerable subjects.

### 5.5. Pets and rodents

Although removing pets from homes is the most advisable, effective long-term strategy to reduce airway responsiveness in asthmatic patients with pet allergy in many studies, it may take at least 20 weeks to decline settled dust as small particles after pet removal (80, 120, 153). There are conflicting results on the effects of pet avoidance in reducing asthma control. HEPA filtration has not been proven to reduce asthma symptoms significantly in people with pet-allergic asthma without pet removals (154). In another study, asthmatic adults, who were sensitized to pets and lived with pets in a shared home, the effect of HEPA was similar (155). Thus, in case of inefficient patient education, allergen interventions, and pharmacotherapy, further studies on allergen immunotherapy should be warranted for pet-allergic patients (156, 157).

The multifaceted integrated pest management intervention (IPM) significantly reduced allergen levels by at least 75% (158). Other approaches (e.g., air purifiers, allergen-proof mattresses, and pillow encasements) may be effectively decrease the concentration of mouse and other animal allergens (159–161). Results from the Inner-City Asthma Study (ICAS) demonstrated that home rodent-specific environmental interventions (e.g., a HEPA air filter placed in the bedroom, filling rodent access points, setting traps, education) reduced mouse allergen levels, asthma-related sleep disorders and limited activities (65). Another randomized trial of the IPM effect in 350 sensitized/exposed asthmatic children showed that prebronchodilator/postbronchodilator forced expiratory flow increased over one year after the mouse allergen concentration was reduced up to 75% (162). In contrast, in The Mouse Allergen and Asthma Intervention Trial (MAAIT) study, 361 mouse-sensitive and exposed children were administered to receive IPM plus pest management education or education alone. There were no significant differences in maximal symptom days between the two groups, although a substantial reduction at 90% of mouse allergen was noted in both groups (163). The lowered level of mouse allergens was associated with fewer hospitalizations and acute care visits (163). Similar findings were demonstrated in other clinical trials (164).

## 6. Conclusion

Much evidence advocates for the strong association between asthma with an allergy to environmental allergens, including outdoor and indoor allergens, particularly in severe asthma. Dust mites, cockroaches, grass, molds, pets, and rodents are the most reported allergens. Allergen avoidance is recommended in managing allergic asthma patients, and several methods have been suggested. However, measures to avoid environmental allergens can be difficult to achieve and require lifestyle modification. Although the clinical effects are inconclusive, environmental allergens avoidance may still benefit patients to some extent, with safe and less adverse effects compared to other pharmacological treatments. Therefore, identifying the sensitized allergens in patients with asthma is crucial, followed by exposure prevention methods. Further trial studies are necessary to elucidate the effectiveness of individually tailored environmental control practices in patients with asthma, specifically those with severe asthma.
